# Sensorimotor control dynamics and cultural biases: learning to move in the right (or left) direction

**DOI:** 10.1098/rsos.160806

**Published:** 2017-02-22

**Authors:** Amanda H. Waterman, Oscar T. Giles, Jelena Havelka, Sumaya Ali, Peter R. Culmer, Richard M. Wilkie, Mark Mon-Williams

**Affiliations:** 1 School of Psychology, University of Leeds, Leeds LS2 9JT, UK; 2 School of Mechanical Engineering, University of Leeds, Leeds LS2 9JT, UK; 3 Bradford Institute of Health Research, Temple Bank House, Bradford Royal Infirmary, Duckworth Lane, Bradford, BD9 6RJ, UK; 4 Norwegian Centre for Vision, The University of South-East Norway, Høgskolen i Sørøst-Norge, Postboks 235, 3603 Kongsberg, Norway

**Keywords:** skill acquisition, culture, sensorimotor control, development, learning

## Abstract

The nativist hypothesis suggests universal features of human behaviour can be explained by biologically determined cognitive substrates. This nativist account has been challenged recently by evolutionary models showing that the cultural transmission of knowledge can produce behavioural universals. Sensorimotor invariance is a canonical example of a behavioural universal, raising the issue of whether culture can influence not only which skills people acquire but also the development of the sensorimotor system. We tested this hypothesis by exploring whether culture influences the developing sensorimotor system in children. We took kinematic measures of motor control asymmetries in adults and children from differing cultures where writing follows opposite directions. British and Kuwaiti adults (*n* = 69) and first grade (5–6 year old) children (*n* = 140) completed novel rightward and leftward tracing tasks. The Kuwaitis were better when moving their arm leftward while the British showed the opposite bias. Bayesian analysis techniques showed that while children were worse than adults, they also showed asymmetries—with the asymmetry magnitude related to accuracy levels. Our findings support the idea that culture influences the sensorimotor system.

## 1. Introduction

Human motor control is notable for its stereotypical nature [1]. For example, ‘reach-to-grasp’ and ‘aiming’ movements show kinematic invariant characteristics within and across individuals [2]. There is widespread support for the hypothesis that such invariant characteristics reflect the development of optimal control processes that underpin movement organization. The presence of sensorimotor invariance appears to provide support for the nativist hypothesis which suggests that universal features of behaviour reflect biologically determined cognitive substrates [3,4]. However, recent research has shown that the cultural transmission of knowledge enables cognitively driven behavioural universals while retaining plasticity at the level of the individual [5]. Indeed, it is a matter of common observation that different individuals learn different skills throughout their lifetime, with the skills acquired by an individual being a function of their surrounding culture. Therefore, cultural traditions may affect the developing sensorimotor phenotype (as optimal control in one culture may not be optimal in another).

The influence of culture on the development of the sensorimotor system has not been well investigated. It is obviously the case that cultural traditions affect the motor system by constraining *which* skills we acquire over the lifespan. But can culture influence the development of the sensorimotor system? There is general consensus that the human motor control architecture includes both adaptive forward models and inverse models. Forward models are used by the central nervous system to estimate the sensory consequences of motor actions while inverse models specify the motor commands appropriate for a desired behaviour [6–8]. The cerebellum has been implicated in forward control [9] and its role has been confirmed by neuroimaging [10] and neuropsychological deficits [11]. This central role of the cerebellum in forward (predictive) control is widely accepted but it is also recognized that forward control is distributed across the cortex, in areas such as the posterior parietal cortex [12]. There is increasing evidence that these predictive mechanisms underpin our ability accurately to perceive the world and create generative models that modulate our thoughts—a viewpoint known as the Bayesian brain hypothesis [13].

Forward models are important for sensorimotor control as they allow the system to predict how our bodies interact with the world. For example, the biomechanics of the human arm mean that different inertias and muscle dynamics are involved when moving the arm leftwards rather than rightwards. It follows that the control dynamics are fundamentally different for leftward movement compared with rightwards (i.e. different directions require different motor commands and produce disparate sensory consequences). Indeed, it has been found that adults from the UK show better performance when moving a hand-held stylus rightwards versus leftwards when tracing novel shapes [14]. Notably, this finding was seen in both the preferred and non-preferred hand of right-handers and left-handers, suggesting the presence of effector-independent rules regarding the control dynamics of hand-held manipulanda. Theorists generally agree that motor control is organized in a hierarchical fashion with movement goals initially specified at an abstract effector-independent level [1]. The fact that a rightward direction bias was seen in both the right and left hand suggests that cultural exposure might have shaped the dynamics of the neural controllers that determine *how* the arms move. In other words, the cerebro-cerebellar system may have developed enhanced state estimation abilities for the control dynamics of the most frequently executed movement type, with culture determining the relative frequency of arm movements to the left versus movements to the right. Thus, if culture influences the developing system, we would expect these systems to develop forward models that are better tuned to performing particular movements in one direction than another.

The fact that arbitrary conventions (e.g. driving on the left) become cultural norms provides a way of testing directly whether culture affects sensorimotor control development. A myriad of different scripts exist across a wide range of cultures, with some traditions evolving reading and writing from left to right (e.g. English) or vice versa (e.g. Arabic), providing a binary contrast between cultural environments. If childhood development involves learning the control dynamics of the arm (i.e. refining forward and inverse models) then cultural traditions of writing direction might be expected to influence motor control, with resultant behavioural asymmetries. In order to explore these ideas, it is necessary to measure the performance of participants on a task that addresses *how* movements are organized rather than describing the *contents* of the acquired skill repertoire (i.e. already learned actions, such as writing the letter ‘a’). We therefore used a novel tracing task with a shape that was unfamiliar to all of the participants but that required the arm to move predominantly leftwards in one condition and rightward in another. This task was, by definition, novel (no participant had ever steered their hand along this path previously) and yet possessed a structure common to many tasks involving arm movement. If cultural pressures influence sensorimotor models then the task structure should result in the appearance of asymmetries.

In terms consistent with the Bayesian brain hypothesis [13], learning the structural relationship between the inputs and outputs of the system across structurally similar motor tasks could decrease the dimensionality of the parameter space when learning a novel task. This allows exploration of the parameter space to be constrained to a region specified by the learnt structure through exploration along a structure-specific meta-parameter (µS) [15]. A forward model may infer the structure between sensory inputs and motor outputs by learning the posterior probability P(S|X) of the structure (S) given the data (X). When learning a new task the meta parameter µS can be inferred by calculating the posterior probability P(µS|S, X) of µS (given S and X). If the forward model has a culturally specific structure, then performance on a novel task that shares the same structure (*S*) should be culturally biased.

## 2. Material and methods

### 2.1. Participants

Forty-eight British adult participants were recruited (21 males and 27 females, age range 18–23, *M* = 20.4; s.d. = 1.2 years; four left-handed). Fifty British children were recruited from a randomly selected English primary school (27 males and 23 females, age range 5.3–6.2, *M* = 5.7; s.d. = 0.30 years; eight left-handed). All British participants spoke English as their first language. Twenty-one Kuwaiti adult participants were recruited (eight males and 13 females, age range 21–45, *M* = 29.0, s.d. = 6.1 years; three left-handed). Ninety Kuwaiti children were randomly recruited from three Kuwaiti primary schools (45 males and 45 females, age range 5.6–6.8, *M* = 6.0, s.d. = 0.30 years; seven left-handed). All Kuwaiti participants spoke Arabic as their first language. Handedness was determined by placing the stylus in a central position in front of the participant and allowing them to pick it up. Minimum sample size was based on previous research investigating structural learning in adults using the same software platform, test methodology, data coding and analysis [14].

British adults were recruited and tested as part of a pedagogical exercise in data collection at the University of Leeds' Psychology Department, which resulted in higher numbers available for testing. The British children were tested via a partnership with a local school, and the Kuwaiti children were tested by agreement with the Kuwaiti Ministry of Education. In both cases the institutions requested that all children within the appropriate age range were tested, which again resulted in different sample sizes. Testing was stopped when either the minimum sample size was reached or when all participants had been tested within the particular University class, or school year groups. The study was approved by the University of Leeds Research Ethics Committee and conducted in accordance with the 1964 Declaration of Helsinki. All participants gave their informed consent before completing the study.

### 2.2. Procedure

**Figure 1.**
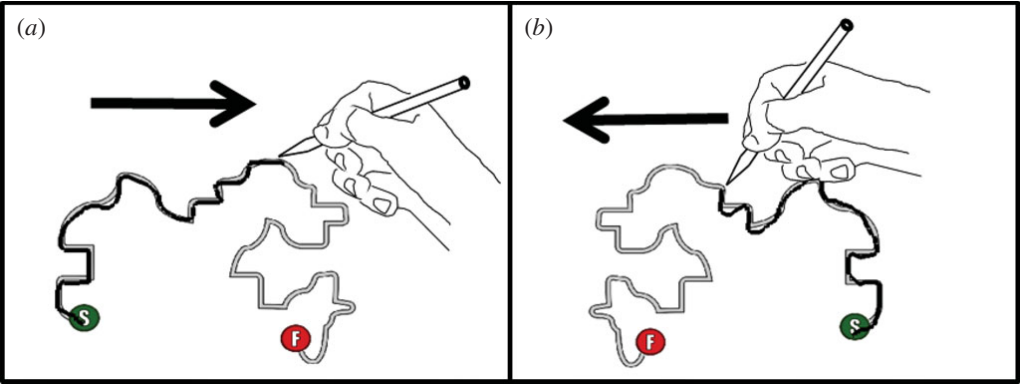
Tracing task used across age and cultural groups. Panel (*a*) demonstrates trials tracing from left to right, and the Panel (*b*) shows tracing from right to left.

All experimental trials were displayed on a tablet PC (Toshiba Portege M700–13P, screen: 260 × 163 mm, 1280 × 800 pixel, 32 bit colour and 60 Hz refresh rate), which was rotated and folded, and positioned on a desk in front of the participant in a landscape position. Participants completed a total of eight experimental trials. Each trial required the participant to trace a shape that appeared on screen using the stylus provided (figure 1) [16].

Participants were instructed to sit in a comfortable position, remove any obstructive items of clothing, and trace the shape on screen from the starting point to the finishing point in one continuous action with their preferred hand. In each trial, the location of the starting point (signified by a green dot) and finish point (signified by a red dot) would differ. Depending on the locations of the starting point in each trial, the directional movement required would differ between tracing from left to right and tracing from right to left. Each trial commenced when participants placed the stylus on the starting point for 500 ms, after which the shape and the finishing point appeared on screen. The participants were then instructed to trace the shape as quickly and as accurately as possible. The trials were repeated with the location of the starting point and finishing point randomized for each participant, therefore requiring the participants to trace the shape four times for each direction.

Kinematic data were collected and processed by specialized software [14,16]. Participants' path accuracy was determined by comparing the movement trajectory against the reference path. A ‘corresponding-point’ technique was used to calculate the error through the trajectory. Point-sets were generated for the input and reference paths by discarding temporal information and resampling the X and Y coordinates at a spatial resolution of 1 mm using linear interpolation. A robust point-registration method [17] was then used to determine the rigid transformation (consisting of translation, rotation and isotropic scaling components) that best transformed the input path to the reference path and identified corresponding points between the reference and input trajectory datasets. A compound dimensional accuracy index was determined by removing any identified translation and evaluating the mean distance between these corresponding points in the original and transformed input point-sets, giving a tracing error score measured in millimetre for each participant.

The results were analysed using Bayesian Estimation techniques. First, a hierarchical linear model was specified as below. The model drew each error score *y_i_* from a normal distribution with a mean that depended on age group, nationality and tracing direction. The standard deviation depended only on age group.

$$\begin{array}{rl} & \beta_{0}∼\text{normal}(1.57,10.0),\\ & \sigma_{m}^{\prime }∼\text{half\_cauchy}(0,3.97),\\ & \beta^{\left(m\right)}∼\text{normal}(0,\sigma_{m}^{\prime }),\\ & \mu_{i}=\beta_{0}+\sum_{1}^{M}\beta^{\left(m\right)}\cdot {\text{X}}^{\left(m\right)}(i),\\ & \sigma ∼\text{half\_cauchy}(0,7.92)\\ \;\text{and}\; & y_{i}∼\text{normal}(\mu_{i},\sigma \cdot {\text{X}}^{\left(1\right)}(i)),\;i=1\ldots N, \end{array}$$

where $\beta^{\left(m\right)}$, *m* = 1,…,*M*, are batches of coefficients. Each $\beta^{\left(m\right)}$ is a vector of deflection parameters for each of the main effects (age group, nationality, tracing direction, subject) and interactions (age group X nationality, age group X tracing direction, nationality X tracing direction, age group X nationality X tracing direction). Within each batch, the coefficients are drawn from a normal distribution with mean 0 and a batch-specific standard deviation (${\sigma_{m}^{\prime }}_{}$). $X^{\left(m\right)}(i)$ is an indicator vector for the *m*-th batch of coefficients for the *i*-th data point. *σ* is a vector of coefficients, where elements *σ*_1_ and *σ*_2_ are the variance parameters for each age group. All priors were chosen to be vague on the scale of the data. Bayes rule was used to estimate the credible values of all the model parameters (*θ*) given the data. The posterior distribution is given by

P(θ | y)∝P(y | θ)P(θ).

A representative sample was drawn from the posterior using the ‘No-U-Turn sampler’ [18] implemented in ‘RStan 2.9.0’. Four chains were started at random values of *θ*, taking 1250 warm-up iterations followed by 1250 samples each. Convergence was visually assessed by examining the chains. All $\hat{R}$ values were below 1.1. Data associated with this work are openly available at the Research Data Leeds Repository [19].

## 3. Results

**Figure 2.**
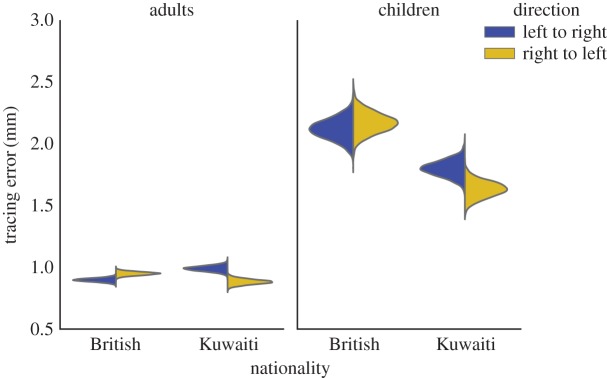
KDE plots of the marginal posterior distributions over the mean tracing error as a function of nationality, age and tracing direction.

The posterior estimates of the condition means are shown in figure 2, which were calculated from the relevant model parameters. Contrasts between these values are reported, which are themselves probability distributions. For each contrast, we report its mean, standard deviation (s.d.) and 95% highest posterior density interval (95% HDI). For a unimodal distribution of mass *M*, the HDI is the narrowest possible interval of that mass [20]. The 95% HDI is the interval in which there is 95% probability that the ‘true’ parameter value falls. This is the property which many researchers erroneously attribute to the frequentist confidence interval [21]. We also report the proportion of the contrast's density that is greater than zero, which we denote as *η*. If *η* > 0.975 or *η* < 0.025 this indicates that the 95% HDI does not contain zero, which is indicated by an asterisk. The contrasts provide distributions over the credible differences between group means. We take a simple Bayesian heuristic approach to testing the ‘null’ hypothesis, where we consider the null unlikely if the contrast's 95% HDI does not contain zero [20]. However, the 95% HDI may still include values which are very close to zero and thus may be considered equivalent to zero for practical purposes. Readers should therefore consider the entire distribution over the contrast, as summarized by its mean and standard deviation, in addition to the 95% HDI.

Tracing error was much lower in the adults than the children (contrast mean = −1.002, s.d. = 0.042, 95% HDI = [−1.085, 0.922], *η* = 0.0*). There was a difference between British and Kuwaiti children with Kuwaitis having lower tracing error (contrast mean = 0.429, s.d. = 0.082, 95% HDI = [0.271, 0.591], *η* = 1*). A small difference between British and Kuwaiti adults was plausible but the 95% HDI contained zero (contrast mean = −0.012, s.d. = 0.019, 95% HDI = [−0.049, 0.025], *η* = 0.245).

British adults showed lower error when tracing in their culturally preferred direction compared with their non-preferred direction (contrast mean = −0.051, s.d. = 0.021, 95% HDI = [−0.093, −0.008], *η* = 0.012*) as did Kuwaiti adults (contrast mean = −0.107, s.d. = 0.031, 95% HDI = [−0.167, −0.046], *η* = 0.001*). For British children, the difference in tracing error for preferred versus non-preferred direction was less clear (contrast mean = −0.051, s.d. = 0.111, 95% HDI = [−0.274, 0.159], *η* = 0.329). The 95% HDI spanned zero and was quite wide indicating uncertainty in the estimate. Kuwaiti children also showed lower tracing error in their culturally preferred tracing direction (contrast mean = −0.168, s.d. = 0.09, 95% HDI = [−0.344, 0.006], *η* = 0.027). The 95% HDI contained zero, but there was a 97.27% chance (1 − *η*) that the contrast was less than zero and the most credible values suggested a difference of reasonable magnitude. We also compared the difference between children and adult performance advantage for their culturally preferred direction (contrast mean = −0.031, s.d. = 0.69, 95% HDI = [−0.177, 0.095], *η* = 0.353). The 95% HDI spanned zero but was also quite wide, making it difficult to establish a null finding.

## 4. Discussion

Clear performance asymmetries were found between adult participants who write and read in opposite directions, demonstrating that cultural traditions influence motor control dynamics. These findings support the idea that population-level behavioural universals can be produced by weak cognitive biases amplified through the effects of culture [5]. The current findings demonstrate that the bias to create predictive sensorimotor models over childhood can influence the development of common forward control processes within a population. Our study also showed asymmetries in the young Kuwaiti children (5–6 years), although there was too much uncertainty in the posterior estimates to establish a difference in the British children. The higher overall accuracy of Kuwaiti children compared with their British counterparts is probably best explained by the fact that Kuwaiti children start formal kindergarten at the age of 3.5 years whereas children in the UK do not enter school until they have passed their fourth birthday [22]. Thus, it is interesting to note that while asymmetries were very likely in the Kuwaiti children, the asymmetry was less clear for the British children. These results suggest that asymmetries arise from mastering the motor control dynamics imposed by cultural constraints.

Our familiarity with cultural norms can mask the implications of this finding. It seems intuitive that individuals from different cultures will have different skill sets—it would clearly be unremarkable if our results had simply showed asymmetries in the ability of the individuals to produce their own scripts. However, the task we employed was entirely novel so participants could not control the movement through previously tuned inverse models—the task required prospective control of the arm dynamics. The important point is that the task was novel but possessed a structure common to other actions learned within a cultural context. All participants were able to carry out the task regardless of movement direction but there was clear evidence that the sensorimotor control processes were different between the populations. The fundamental nature of these differences is emphasized by the observation of these asymmetries in young children. These findings highlight that the observed differences may reflect differences in the ontogeny of sensorimotor control.

The fact that individuals from different reading and writing traditions also show differences in number representation supports the importance of considering culture when attempting to understand human behaviour. For example, the Spatial-Numerical Association of Response Codes (SNARC) effect describes the phenomenon whereby individuals represent numbers according to their normal cultural organization [23]. Thus, smaller numbers are represented on the left side of space in adults from a Western tradition but those from an Arabic culture show the opposite pattern [24]. Notably, individuals from a Western culture typically start counting with the left hand and associate the number one with their thumb when counting on their fingers. By contrast, individuals raised in a Middle Eastern culture prefer to start counting with the right hand and typically associate the number one with their little finger [25]. The implication is that the mechanisms that underpin the processes of cognition (broadly construed) are not innate and invariant human universals. Rather, our thought processes are influenced by cultural norms. In short, our findings support the conjecture that humans are the product of their environment with the cultural milieu affecting our behaviour.

With this in mind our findings may have implications for educational systems. Owing to increased multicultural environments across many countries, children can be exposed to one type of script at home (e.g. right to left), and another when they start formal schooling (e.g. left to right). It is possible that learning two different control dynamics will be advantageous (giving greater sensorimotor flexibility) but it may also cause negative interference. The probability of an advantageous outcome may reflect the richness of the different learning environments, which might mean that other factors, such as socioeconomic position, play a mediating role. These notions are speculative but our experimental findings suggest they are issues that warrant further investigation. In conclusion, our findings support the idea that culture influences the motor system in the same way that culture influences the core cognitive processes involved in the representation of abstract concepts. It follows that a complete understanding of human behaviour requires a consideration of motor control processes, cognitive representations, cultural transmission, and how these various factors interact.

## Data accessibility

Data are available via the Research Data Leeds Repository: http://doi.org/10.5518/148.
