# Correction to ‘Sensorimotor control dynamics and cultural biases: learning to move in the right (or left) direction’

**DOI:** 10.1098/rsos.210231

**Published:** 2021-02-24

**Authors:** Amanda Waterman, Oscar Giles, Jelena Havelka, Sumaya Ali, Peter Culmer, Richard Wilkie, Mark Mon-Williams

*R. Soc. Open Sci*. **4**, 160806. (Published 22 February 2017). (doi:10.1098/rsos.160806)

The author affiliations should read as follows. The original paper has now been updated.

Amanda H. Waterman^1,*^, Oscar T. Giles^1^, Jelena Havelka^1^, Sumaya Ali^3^, Peter R. Culmer^2^, Richard M. Wilkie^1^ and Mark Mon-Williams^1,4,5^

^1^School of Psychology, and

^2^School of Mechanical Engineering, University of Leeds, Leeds LS2 9JT, UK

^3^The Public Authority for Applied Education and Training, PO Box 23778, Safat 22081, Kuwait

^4^Bradford Institute of Health Research, Temple Bank House, Bradford Royal Infirmary, Duckworth Lane, Bradford BD9 6RJ, UK

^5^Bradford Norwegian Centre for Vision, The University of South-East Norway, Høgskolen i Sørøst-Norge, Postboks 235, 3606 Kongsberg, Norway

*Corresponding author

